# A protocol for isolation and identification and comparative characterization of primary osteoblasts from mouse and rat calvaria

**DOI:** 10.1007/s10561-019-09751-0

**Published:** 2019-03-18

**Authors:** Baoyan Liu, Yanqin Lu, Yong Wang, Luna Ge, Naixiang Zhai, Jinxiang Han

**Affiliations:** 1 School of Medicine and Life Science, Shandong Academy of Medical Sciences, University of Jinan, 18877, Jingshi Road, Jinan, 250012 Shandong China; 2 Key Laboratory for Rare Disease Research of Shandong Province, Shandong Medical Biotechnological Center, Shandong Academy of Medical Sciences, 18877, Jingshi Road, Jinan, 250012 Shandong China; 3 Department of Neurosurgery, Shandong Cancer Hospital affiliated to Shandong University, 440 Jiyan Road, Jinan, 250114 Shandong China

**Keywords:** Mouse, Rat, Calvaria, Osteoblast, Comparative

## Abstract

Calvaria from neonatal mouse and rat is ideal resource for osteoblasts but can be easily contaminated by other cells such as fibroblasts. Here, we established a protocol for isolation and purification of primary osteoblast by enzyme sequential digestion and differential adhesion. In addition, we compared the phenotypic and functional traits of osteoblasts from mouse and rat which are commonly employed in studies. The method applied equally to rat and mouse in osteoblasts isolation and was corroborated its feasibility and validity. The results also provided us evidences for other experiments such as choosing a certain time point to give intervention and do the relevant tests.

## Introduction

Osteoblasts are responsible for bone formation which is accompanied by bone resorption accomplished by osteoclast in keeping bone metabolic balance. Its proliferation and differentiation play a key role in osteogenesis. Mesenchyme stem cells (MSCs) and osteoblasts are suitable for bone tissue regeneration for their capacity of bone matrix synthesis, secretion and mineralization in bone formation. Microstructured Ti implants containing osteoblast were used to regulate bone formation to determine if sexual dimorphism affected their responses to systemic hormones during implant osseointegration (Berger et al. 2018). Previous researches have shown that osteoblast differentiation can be affected by osteoclast and MSC and adipocyte. For example, coculture of osteoblast with these cells can mimic in vivo environment to investigate their interaction through released cytokins and autocrine and paracrine factors (Malekshah et al. 2006). Osteoblast differentiation was regulated by some factors which were released during bone resorption to ensure the new bone formation (Cao 2011). MSCs repress osteoblast differentiation under osteogenic-inducing conditions (Santos et al. 2015). Thus, it makes sense to make clear the relationship between osteoblast with other cells and through what factors they function in bone formation.

The earliest isolation of primary bone cells from rat, considered as osteoblasts or osteocytes for presence of considerable alkaline phosphatase in the cytoplasm, was successfully carried out by Peck et al. (1964) though overgrowth of fibroblasts in the culture and no mineralization nodules were observed. Afterwards, Wong and Cohn provided a basal procedure for obtaining spindle-shaped cells with an osteoblastic phenotype over extended culture (1974). Here, we use enzyme sequential digestion to remove most osteo-chondrogenic progenitors existing in periosteum. There are a small number of osteocytes in calvaria of neonatal mouse or rat and the number will gradually decline because of its limited proliferative activity. With subculturing and differential adhesion, spindle-shaped osteoblasts become the main component of the adherent cell population.

Saos2 cells, MG-63 and MC3T3-E1 and other cells are usually used in the osteoblast related research. Although these cells share the similar osteoblastic phenotype, the functional activity of osteosarcoma cells and their response to osteoblast regulatory factor exhibit heterogeneity. Therefore, primary osteoblasts from mouse or rat are still ideal cells to a large extent for mimicking internal process especially in genetically modified animals. Endogenous and exogenous cytokines and microRNAs play a role at certain stage and target specific genes. It has been revealed that surface roughness of Ti disks promotes osteogenic differentiation of less mature cells. Thus, it is necessary to illuminate the expression pattern of phenotypic maker at different stages during osteoblast differentiation (Lohmann et al. 2000).

Previous papers have described the methods of primary osteoblasts isolation from calvaria (Jonason and O’Keefe 2014). However, there still exist many questions, such as other cells like fibroblast and chondrocyte and osteocyte contamination. Whether this method is suitable for rat and what are the differences of gene expression and capacity of mineralization between osteoblasts from rat and mouse have not been elaborated.

Therefore, this paper showed a detailed and standard protocol for isolation and purification and identification of primary osteoblast from mouse and rat calvaria. In addition, the expression profile of gene marker and the comparative characterization of primary osteoblasts from mouse and rat were described in this study.

## Methods and materials

All animal procedures were performed under the approval of the Committee of Ethics in Animal Research of the University of Jinan. Mouse and rat born within 48 h were obtained from Shandong University Laboratory Animal Center. We use mouse as example to illustrate isolation and purification of osteoblasts in the following experiments.

Primary osteoblast isolation and purification from mouse and rat calvaria

1. Choose mouse and rat born within 48 h for the experiment, and immerse the mouse with 75% (vol/vol) alcohol for 10 min to clean the body surface.
2. Kill mouse by cervical dislocation and pull down the head skin and dissect calvarium. Then remove periosteum and blood vessels and interstitial cartilage attached to the bone to reduce other cells. Besides, Wash the calvarium with Hanks solution three times to deplete blood cells. All of these operations were under sterile condition.
3. Add 0.25% trypsin containing 0.02% EDTA to the bone chips for 25 min after it was excised into approximate 1–2 mm^3^ pieces at 37 °C to digest fibrous tissue such as periosteum.
4. Digest bone chips in 5 ml Hanks solution containing 0.1% (wt/vol) Collagenase I and 0.05% trypsin containing 0.004% EDTA for 1 h in a shaking incubator at 37 °C with a shaking speed of 200 r.p.m.
5. Collect the released cells and discard digest medium by centrifugation for 8 min at 1000 r.p.m. Suspend the cells in 5 ml of α-MEM (containing 1 g/L d-Glucose and l-Glutamine, BI, USA) containing 10% (vol/vol) FBS and transfer the cells to 25 cm^2^ plastic culture flask (polystyrene cell culture flask, Nest, China). Incubate the culture flask at 37 °C in a 5% CO_2_ incubator and subculture cells when reach 80% fusion.
6. Culture the digested cells at 37 °C for 20 min before transferring the non-adherent cells to another culture flask. Repeat this step for two times.
  Note: this step aims to remove most fibroblasts because fibroblasts are easier to adhere to plastic flask than osteoblasts.
7. Change the culture medium every 2 days. The cells at passage 3–5 were used in the subsequent experiments.

### Cell proliferation assay

Cells isolated from calvaria were trypsinized when confluence and then reseeded on 24-well plate (round wells) at a density of 5000 cells per well. Population doubling time was defined as the period from seeding to passaging cells at a split ratio of 1:2 when reaching 100% fusion. Population doubling time was observed across 5 passages. The medium was changed every 3 days throughout the whole experiments. In addition, cells at passage 2 were seeded on 96-well plate at a density of 1000 cells per well to evaluate cell proliferation with Cell Counting Kit-8(CCK-8) for 12 h, 24 h, 48 h, 72 h. Briefly, cells were added with CCK-8 at a specific point in time and then incubated at 37 °C for 1 h and then the optical density was read at 450 nm. The data of absorbance were directly proportional to the number of proliferating and living cells.

### Alkaline phosphatase staining for alkaline phosphatase activity

Cells at passage 4 were seeded at 2 × 10^4^ per well in 24-well plates (round wells). Alkaline phosphatase staining was followed by the kit instruction at different points during culture with and without osteoblast induction medium such as 0 day, 3 days, 7 days, 10 days and 15 days. The brief staining steps are as follows:

1. Discard the medium before washing cells with GENMED reagent A and fix cells with regent B.
2. Add staining working liquid and incubate at room temperature in dark for 30 min.
3. Aspirate working liquid and wash the cell surface with regent A and then remove it.
4. Cells with alkaline phosphatase activity were dyed purple-black or blue-black.

### Alkaline phosphatase activities assay with p-NPP as alkaline phosphatase substrate

Alkaline phosphatase activities were evaluated with p-NPP as ALP substrate. Like ALP staining experiments, cells at passage 4 were seeded at 2 × 10^4^ per well in 24-well plates and then ALP activities were tested at different points during culture with and without osteoblast induction medium such as 0 day, 3 days, 7 days, 10 days and 15 days. More specifically, cells were washed with Hanks and then cells were lysed with cell lysis buffer (Tris-HCl 25 mM,TritonX-100 0.5%) at 4 °C for 2 h. After complete lysis, 100 ul p-NPP was added to 50 ul cell lysate and the mixture were incubated in 37 °C for 20 min, meanwhile, 5ul cell lysate were used for protein quantification with enhanced BCA protein assay kit (Beyotime,China).The absorbance at 405 nm was detected by spectrophotometric method. ALP activities were normalized by protein concentrations.

### Alizarin red S staining and quantification for mineralization nodules assay

Cells at passage 4 were seeded at 2 × 10^4^ per well in 24-well plates (round wells). Alizarin red S staining was carried out at different points during culturing with and without osteogenic induction medium such as at 0 day, 3 days, 7 days, 10 days and 15 days. The brief staining steps are as follows:

1. Wash cells three times in 1 × PBS.
2. Fix cells with 4% paraformaldehyde for 30 min and remove it.
3. Add 500 μl Alizarin red S staining solution to cover the cell surface and incubate at room temperature from light for 20 min.
4. Discard Alizarin red S solution and wash cells with 1 × PBS to remove excessive staining solution.
5. Dry water and observe staining results.

To quantitatively evaluate the mineralization nodules, the nodules were dissolved in 500 μl 10% cetylpyridinium choride at 37 °C for 1 h and then the absorbance at 570 nm was measured.

### Osteoblasts induced with and without osteogenic induction medium (OIM) and real-time quantitative PCR(RT-qPCR) analysis for gene expression

1. Seed passage 4 cells at a density of 2 × 10^4^ per well into 24-well plates and divide into two groups including OIM (osteogenic induction medium) and Control group.
2. Culture cells with OIM (αMEM, 10%FBS, 50 mg/L ascorbic acid and 10 mMβ-glycerol phosphate) and Control medium (αMEM, 10%FBS) for varying duration.
3. Extract RNA from two groups with trizol reagent according to the instruction.
4. Perform real-time quantitative PCR detection with 10 μl reaction mixture (FastStart Universal SYBR Green Master(Rox) 5 μl, cDNA 20 ng, Forward Primer 0.5 μl at concentrations of 10 pmol/μl, Reverse Primer 0.5 μl at concentrations of 10 pmol/μl, Rnase free water). Glyceraldehyde 3-phosphate dehydrogenase (GAPDH) was employed as reference gene for normalization. Primers used in this study were shown in the Table 1.

**Table 1:** Primer sequences for the RT-qPCR analysis

Genes	Forward primers	Reverse primers	Species
ALP	AATGAGGTCACATCCATCCTG	CACCCGAGTGGTAGTCACAA	Mouse
OCN	GCGCTCTGTCTCTCTGACCT	TTCAGGAGGGTAGTTACCCAAA	Mouse
RUNX2	ACAGAGCTATTAAAGTGACAGTGGAC	GGCGATCAGAGAACAAACTAGG	Mouse
Col1a1	CATGTTCAGCTTTGTGGACCT	GCAGCTGACTTCAGGGATGT	Mouse
GAPDH	AAGAGGGATGCTGCCCTTAC	CCATTTTGTCTACGGGACGA	Mouse
ALP	ACGAGGTCACGTCCATCCT	CCGAGTGGTGGTCACGAT	Rat
OCN	GGACATTACTGACCGCTCCT	TTTTCAGTGTCTGCCGTGAG	Rat
RUNX2	CCACAGAGCTATTAAAGTGACAGTG	ACAAACTAGGTTTAGAGTCATCAAGC	Rat
Col1a1	GCATGGCCAAGAAGACATCC	CCTCGGGTTTCCACGTCTC	Rat
GAPDH	TGGGAAGCTGGTCATCAAC	GCATCACCCCATTTGATGTT	Rat

### Statistical analysis

**Fig. 1:**
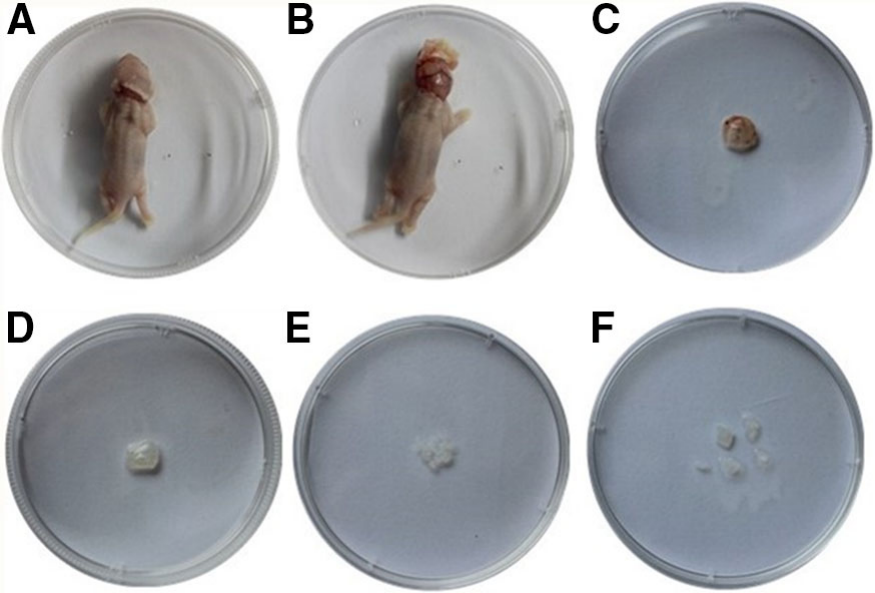
Isolation of calvaria from mouse. Mouse was killed by cervical dislocation. **A** Cut off the neck skin. **B** Pull down the head skin. **C** Take off calvaria. **D** Wash the calvarium with hank’s buffer solution three times to deplete blood cells. **E** Remove periosteum and blood vessels and interstitial cartilage. **F** Excise into approximately 1–2 mm^3^ pieces. Isolation of calvaria from rat was in the identical method

All data were expressed as mean ± standard deviation. A two-tail Student’ test was used for comparing two independent groups. *P* < 0.05 was established as statistically significant difference. GraphPad Prism 4.0 was employed in illustration (Fig. 1).

## Results

### Cell proliferation rate varied between mouse and rat

**Fig. 2:**
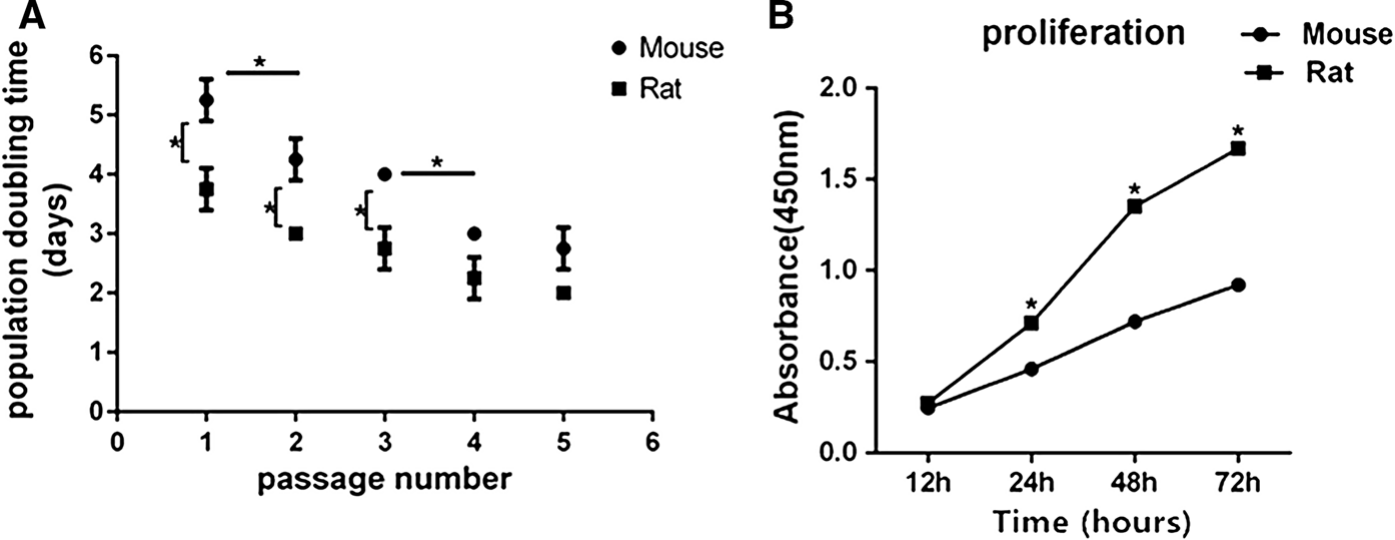
Expansion rate of osteoblast from Mouse and Rat. **A** Cells were passaged at a split ratio of 1:2, population doubling time shown across 5 passages. **B** Cells proliferation at passage 2 was assayed with CCK-8 and absorbance at 450 nm was examined for 12 h, 24 h, 48 h, 72 h. *Indicated significant difference between two groups at the same time point. The data were presented as the mean ± standard deviation (n = 3)

In mouse and rat osteoblasts, time throughout two passages which indicated growth rate both showed decline from isolation to passage 5 (Fig. 2A). Expansion rate increased more significantly in passage 2 than in passage 1 and the population doubling time also decreased more significantly in passage 4 than in passage 3 by approximately 1.5-fold. Moreover, cells proliferation at passage 2 assayed with CCK-8 was lower in mouse osteoblasts than in rat osteoblasts when cultured in control medium for 24 h, 48 h and 72 h (Fig. 2B). Doubling time of two species at the same time point increased at passage 1 by approximately 1.6-fold and at passage 2 by approximately 1.5-fold and at passage 3 by approximately 1.6-fold in mouse osteoblast than in rat osteoblast. In passage 4 and 5, mouse osteoblasts were not statistically significant different from rat osteoblasts.

### Alkaline phosphatase activity

**Fig. 3:**
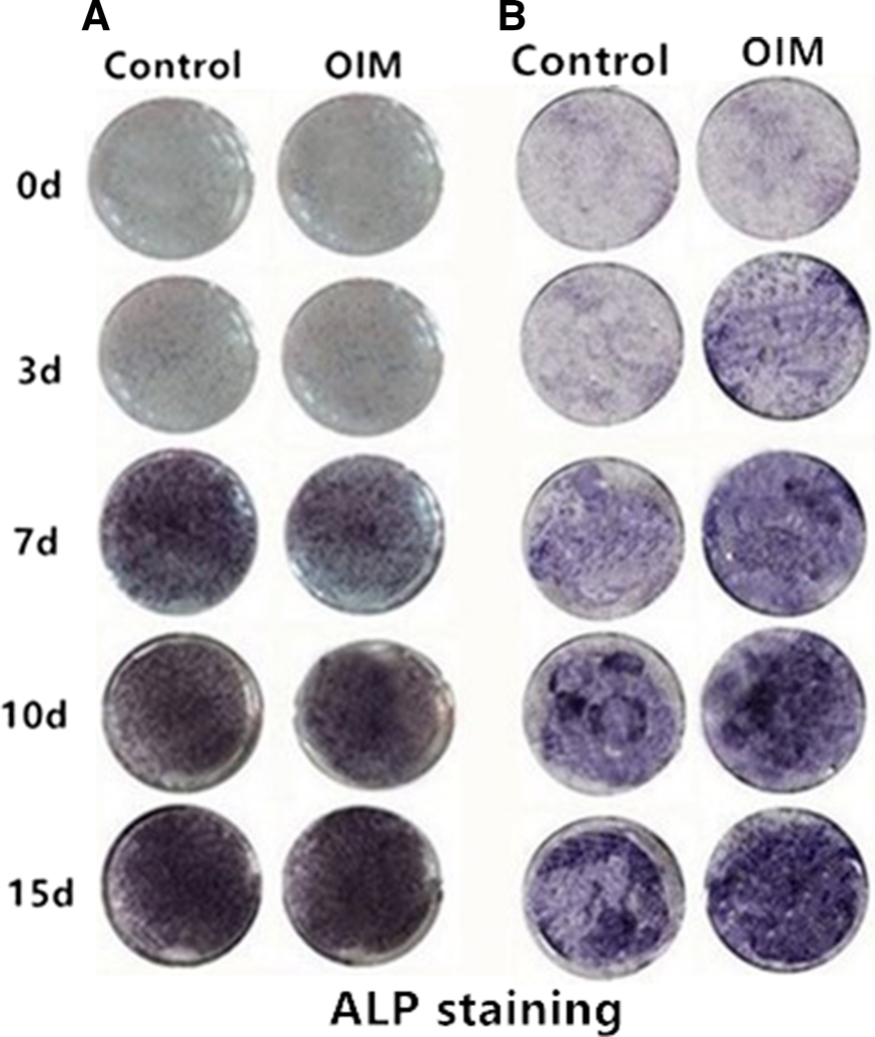
ALP staining for osteoblast cultured with and without OIM at different time points. **A** ALP staining of osteoblast from mouse calvaria when cultured in OIM and control medium for 0 day, 3 days, 7 days, 10 days and 15 days. **B** ALP staining of osteoblast from rat calvaria when cultured in OIM and control medium for 0 day, 3 days, 7 days, 10 days and 15 days. **C**, **D** ALP activity of osteoblast from mouse and rat calvaria when cultured in OIM and control medium at particular points in time. **E** ALP activity of osteoblast from mouse and rat calvaria when cultured in OIM at particular points in time. *Indicated significant difference between two groups at the same time point. The data were presented as the mean ± standard deviation (n = 3)

**Fig. 4:**
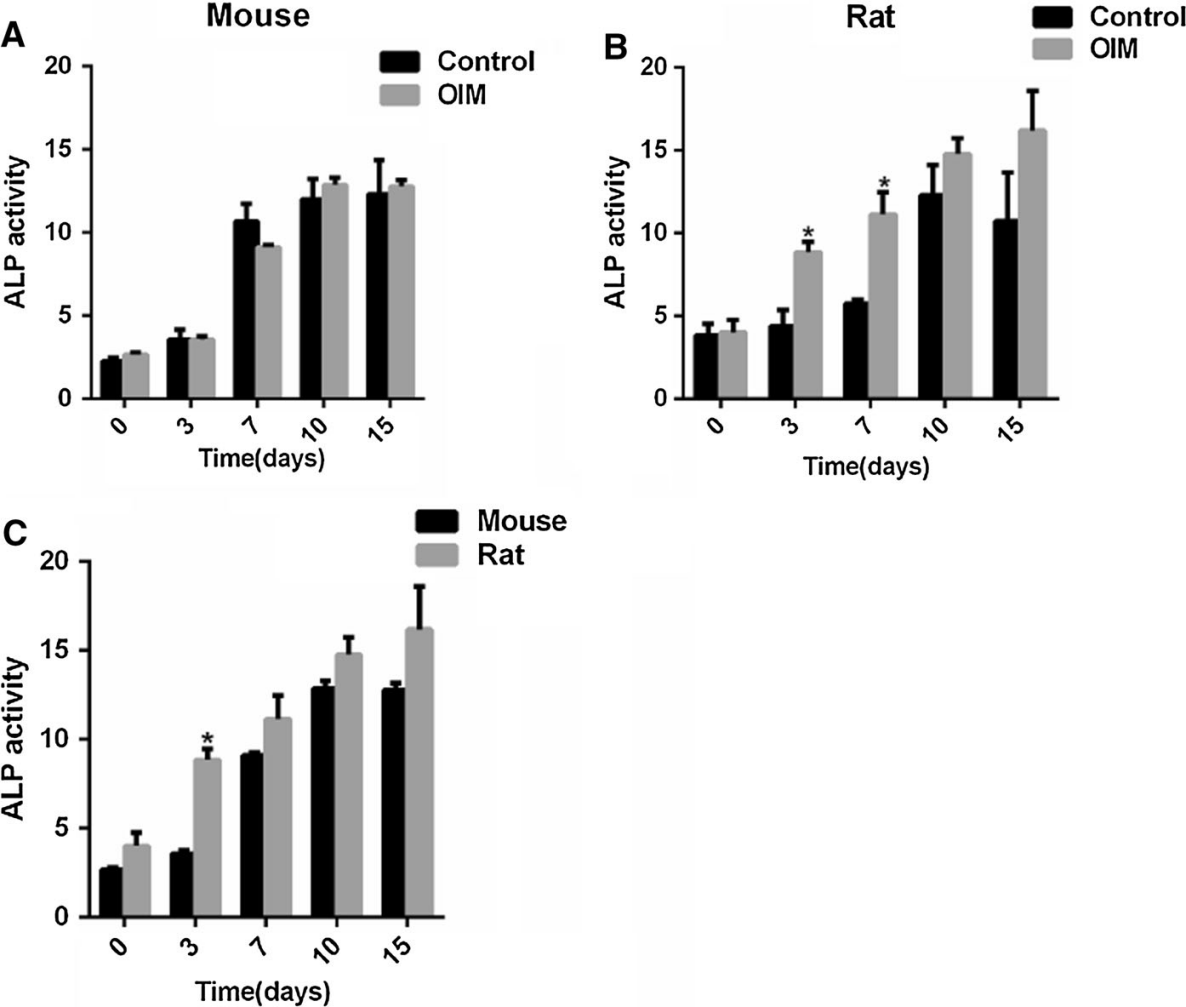
ALP activity of osteoblast cultured with and without OIM at different time points. **A**, **B** ALP activity of osteoblast from mouse and rat calvaria when cultured in OIM and control medium at particular points in time. **C** ALP activity of osteoblast from mouse and rat calvaria when cultured in OIM at particular points in time. *Indicated significant difference between two groups at the same time point. The data were presented as the mean ± standard deviation (n = 3)

Mouse and rat osteoblast were both cultured with and without OIM. With culturing time extending, mouse and rat osteoblasts both showed increased alkaline phosphatase activity (Fig. 3A, B). For example, the ALP activity of rat osteoblasts cultured in OIM for different days were as follows: 4.01 ± 0.76 for day 0, 8.85 ± 0.61 for day 3, 11.15 ± 1.31 for day 7, 14.77 ± 0.97 for day 10, 16.19 ± 2.42 for day 15 (Fig. 4B). In mouse osteoblasts, there were no obvious differences between control medium and OIM (Fig. 4A), but alkaline phosphatase activity increased more significantly in osteoblasts cultured in OIM (8.85 ± 0.61 for day 3, 11.15 ± 1.31 for day 7) than in control medium (4.39 ± 0.97 for day 3 and 5.75 ± 0.26 for day 7) in rat (Fig. 4B). Having analyzed ALP activity of mouse osteoblasts and rat osteoblasts separately from control medium and OIM, we found at the early stage, rat osteoblasts showed higher ALP activity (8.86 ± 0.61) than mouse osteoblasts (3.20 ± 0.2) in OIM on day 3 (Fig. 4C). However, ALP activity of mouse osteoblasts exceeded rat osteoblasts from day 7 independent of cultured in control medium and OIM (Fig. 4C).

### Mineralization capacities

**Fig. 5:**
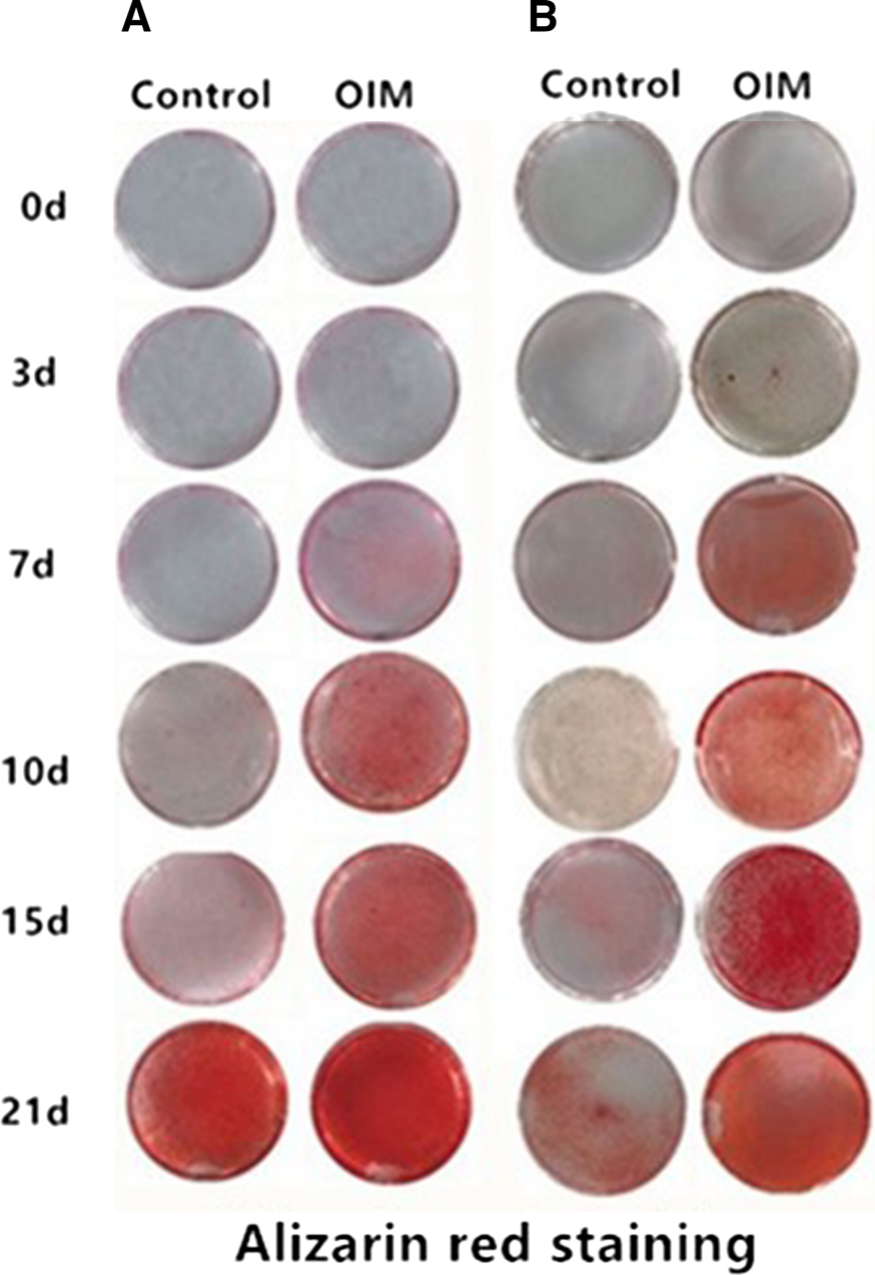
Alizarin red staining for osteoblast cultured with and without OIM at different time points. **A** Alizarin red staining of osteoblast from mouse calvaria when cultured in OIM and control medium for 0 day, 3 days, 7 days, 10 days, 15 days and 21 days. **B** Alizarin red staining of osteoblast from rat calvaria when cultured in OIM and control medium for 0 day, 3 days, 7 days, 10 days, 15 days and 21 days

**Fig. 6:**
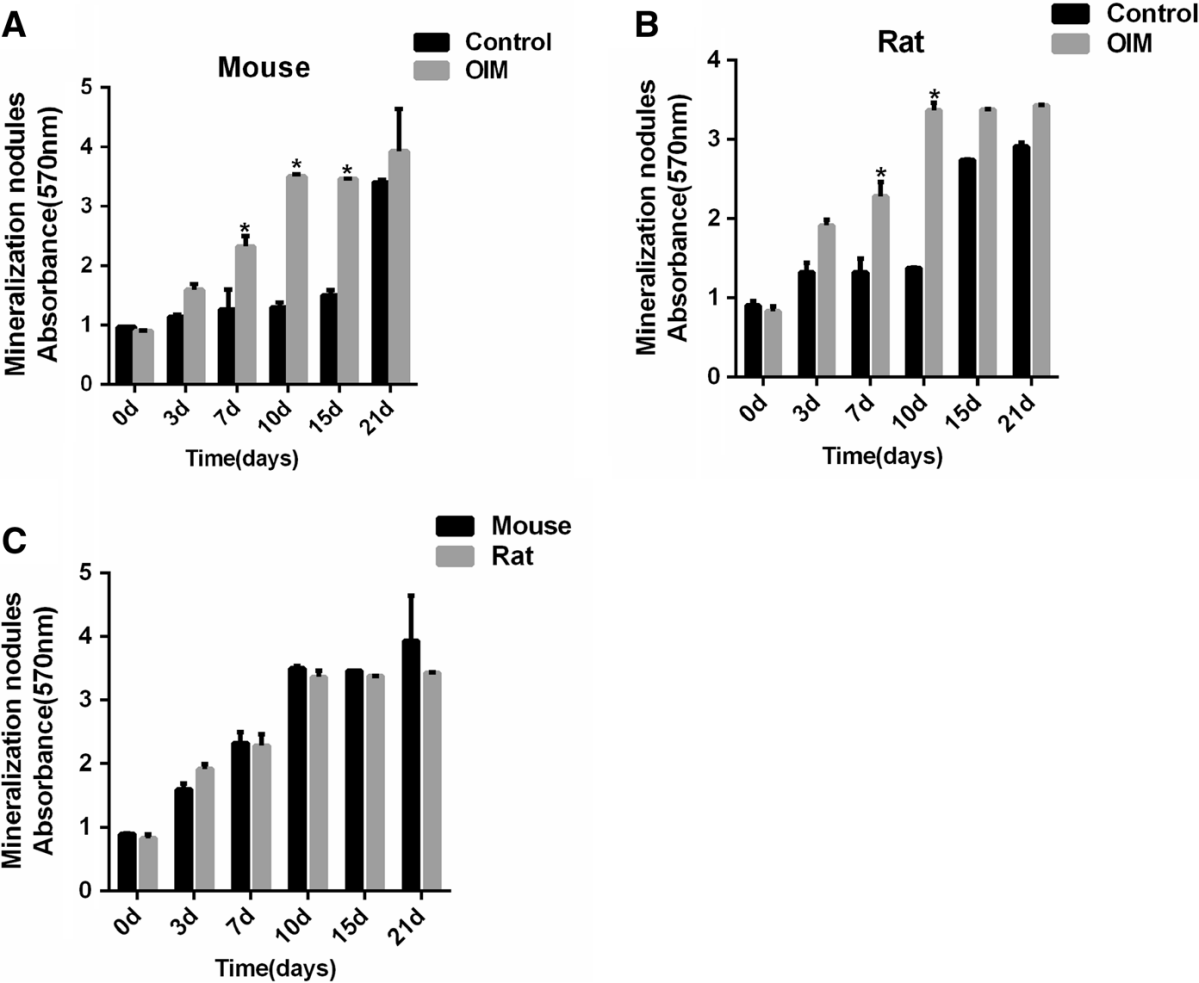
Quantitative analysis for mineralization nodules. **A**, **B** Absorbance at 570 nm for osteoblasts from mouse and rat calvaria when cultured in OIM and control medium at particular points in time. **C** Absorbance at 570 nm of mineralization nodules for osteoblasts from mouse and rat calvaria when cultured in OIM at particular points in time. *Indicated significant difference between two groups at the same time point. The data were presented as the mean ± standard deviation (n = 3)

In the same control medium, osteoblasts derived from mouse and rat form mineralization nodule from approximately day 10, but in OIM, the time for nodules formation was advanced in both osteoblasts isolated from the two species (Fig. 5A, B). Under osteogenic induction conditions, nodules of mineralization appear on day 3 in rat osteoblasts which was earlier than mouse osteoblasts on day 7. Analysis of nodules amount from mouse and rat osteoblasts revealed no statistically significant differences between them from day 10. The absorbance at 570 nm of mineralization nodules were as follows: 3.51 ± 0.04 on day 10, 3.37 ± 0.004 on day 15 and 3.93 ± 0.71 on day 21 in mouse osteoblasts, 3.37 ± 0.09 on day 10, 3.38 ± 0.003 on day 15 and 3.43 ± 0.006 on day 15 in rat osteoblasts (Fig. 6C).

### Bone related gene revealed time-dependent upregulation under osteogenic induction

**Fig. 7:**
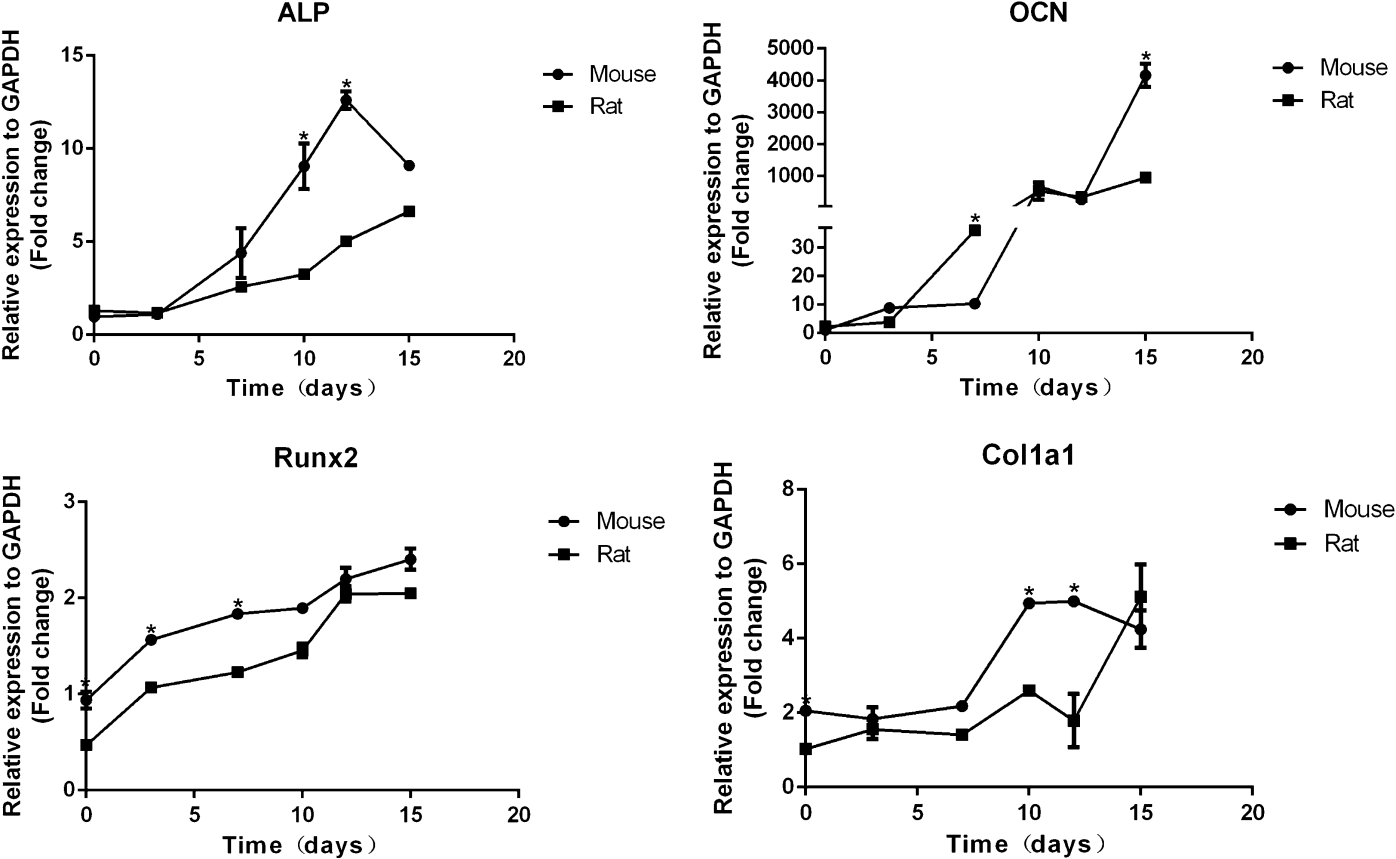
Expression of bone related genes by RT-qPCR. The expression of ALP, OCN, Runx2 and Col1a1 in the indicated time after osteogenic induction in mouse and rat. First the cp value of gene expression was normalized to the expression of GAPDH. Then the data presented in the diagram was the expression of gene normalized to the expression of the gene in mouse osteoblasts at day 0. *Indicated significant difference between two groups at the same time point. The data were presented as the mean ± standard deviation (n = 3)

Under the same conditions, Osteocalcin (OCN) expression increased with the induction as well as Runt-related transcription factor-2 (Runx2) and Col1a1 both in mouse osteoblasts and rat osteoblasts (Fig. 7). Compared with rat osteoblasts, ALP expression was lower but not statistically significant in mouse osteoblast at the early stage (*P *= 0.06); but after 3 days, it became higher, which was consistent with expression of Runx2 and Col1a1.In mouse osteoblasts, ALP expression decreased on day 15 compared with day 12 under osteogenic induction (*P *< 0.05). When compared with the other three genes, OCN showed a more rapid time-dependence and up-regulation by approximately hundreds of times both in mouse and rat osteoblasts compared with the expression in mouse osteoblasts at day 0(*P *< 0.05). The expression of Col1a1 showed a narrow range over the time of induction both in mouse and rat.

## Discussion

In this paper, we provide an easy method for isolation and purification of osteoblast applying to mouse and rat which are usually employed in researches. Spindle-shaped osteoblasts were obtained after subculture and differential adhesion. Cells derived from mouse and rat calvaria were proved to be osteoblast as we observed their ALP activity and mineralization capacities with or without OIM. In addition, the results from a side confirmed the feasibility and effectiveness of our protocol. Spontaneous osteogenesis is a characteristic of osteoblast even without osteoblast induction medium. The osteogenesis process can be greatly accelerated when given osteogenic induction.

Passage 4 osteoblasts were used in the experiment for their rapid expansion rate and high purity. It takes longer time for primary cells derived from calvaria both of mouse and rat to passage, which may be a result of a mixture of other cells with lower proliferative capacity. As the purity increased, the proliferation rate of osteoblast was enhanced. Even the method for mouse osteoblast isolation and characteristics has been performed in other study [8]. However, no work about the differences between the two species has been reported.

Alkaline phosphatase as early phenotype marker of the osteoblast, its enzyme activity increases as osteoblasts mature and decreases as mineralization starts, which is an indicator of late differentiation. Whether under osteogenic induction or not, ALP activity was higher in rat osteoblast than mouse osteoblast before day 3. However, the ALP activity of mouse was much higher than rat since the early stage. Mineralization of rat osteoblast initiated earlier than mouse osteoblast but the degree of mineralization was lower than mouse osteoblast either under osteogenic induction or not. Cell differentiation is characterized by osteocalcin production and mature phenotype is evidenced by high level of alkaline phosphatase activity and OCN expression. Osteocalcin is well known as secreted solely by mature osteoblasts and can strongly bind to hydroxyapatite crystals in the mineralized matrix as carboxylated (Hauschka et al.^1989^). Consistent with ALP activity and expression, rat osteoblast expressed higher level of OCN in OIM, compared with mouse osteoblast. Runx2 plays a key role in osteoblast differentiation and skeletal morphogenesis (Lohmann et al. 1997; Otto et al.^1997^) and is essential for the expression of several osteoblast marker genes such as osteocalcin (OCN) and collagen I (ColI) (Ducy et al. 1997; Zaidi et al. 2001). Col1a1 and Col1a2 encode two alpha chains of type I collagen as a major component of the bone matrix (Pollitt et al. 2006). OCN and Col1a1 expression increased with induction time. The trend of the relative expression of the OCN is generally consistent with Col1a1 in mouse and rat osteoblasts. This is because collagen I construct the building block and OCN bind hydroxyapatite crystals construct the filler and structural support of bone matrix. They are crucial in the formation of organic bone matrix. Mouse Col1a1 expression levels is higher than rat at the same time point. In both species, OCN expression showed a sharp up-regulation from 10 days. To be more concrete, mouse OCN expression is similar to rat at 10 days, whereas at 15 days expression is higher than rat.

Specific genetic characteristics are shown among species, which make them more suitable to a particular study. For example, the pattern of LRRK2 expression significantly related to Parkinson’s disease (PD) differs in distinct neuronal subtypes between rats and mice (West et al. 2014). In this study, we conducted a comparative analysis of osteoblast related phenotypic and functional traits and patterns in the same way. Thus, we not only provide an effective method for osteoblast isolation and purification, but also useful information as to the bone formation process independent of mouse or rat used.

## Conclusion

We provide a protocol for osteoblast isolation and purification applying to mouse and rat. Spindle-shaped osteoblasts with the capacity of mineralization can be obtained by the method. However, there are significant differences in cell expansion and functional and genetic traits between mouse osteoblasts and rat osteoblasts. Based on the elaboration of characteristics during bone formation, this paper would provide clues to the selection of mouse and rat in osteoblast related studies.
